# Late-onset ornithine transcarbamylase deficiency with neurological damage and serial brain multimodality monitoring: a case report

**DOI:** 10.3389/fmed.2026.1819409

**Published:** 2026-07-01

**Authors:** Yujie Zhou, Yiting Liu, You Zhou, Yunxing Cao

**Affiliations:** 1 Department of Critical Care Medicine, The Second Affiliated Hospital of Chongqing Medical University, Chongqing, China; 2 Department of Critical Care Medicine, Chongqing General Hospital, Chongqing, China

**Keywords:** brain multimodality monitoring, intensive care unit, late-onset ornithine transcarbamylase deficiency, paroxysmal sympathetic hyperactivity, targeted temperature management

## Abstract

**Background:**

Late-onset ornithine transcarbamylase deficiency (OTCD) is a rare urea cycle disorder that may present in adulthood with acute hyperammonemic encephalopathy and cerebral edema. The disease has a rapid onset and progression, resulting in high mortality and poor prognosis.

**Case presentation:**

This case report describes a patient with late-onset OTCD who presented to the Department of Critical Care Medicine at Chongqing Medical University Second Affiliated Hospital in August 2024. The patient presented with altered consciousness, generalized convulsions, severe hyperammonemia, and diffuse cerebral edema. He received continuous renal replacement therapy (CRRT), ammonia-lowering therapy, mechanical ventilation, antiepileptic treatment, cerebral edema-directed care, and targeted temperature management (TTM). Serial electroencephalography (EEG), transcranial Doppler ultrasonography (TCD), near-infrared spectroscopy (NIRS), and neuroimaging were used to assess neurological status and cerebral physiological changes over time. Although the blood ammonia level normalized early, the patient subsequently developed fluctuations in vital signs. This suggests that, in patients with OTCD, ongoing or progressive brain injury should still be considered even after ammonia levels have returned to the normal range. The results of multimodal neuromonitoring were used together with the clinical status and neuroimaging findings for continuous reassessment. This approach provided bedside information that supported early recognition of clinical changes and adjustment of the treatment strategy.

**Conclusion:**

Ornithine transcarbamylase deficiency is rare and remains difficult to diagnose clinically. This case suggests that delayed neurological deterioration may still occur in late-onset OTCD even after biochemical improvement. Multimodal neuromonitoring may provide useful bedside information for individualized neuroprotective management and may help guide treatment adjustment and the differential diagnosis of possible complications.

## 1 Introduction

Ornithine transcarbamylase deficiency (OTCD) is an inherited metabolic disorder of the urea cycle caused by deficient activity of ornithine transcarbamylase. The reported incidence of OTCD ranges from 1 in 80,000 to 1 in 56,500 (1), and approximately 70% of patients present with late-onset disease (2). The timing of presentation and clinical severity varies according to residual enzyme activity and catabolic or environmental triggers. Initial manifestations are often nonspecific, including vomiting, behavioral change, confusion, and reduced oral intake, which may delay diagnosis and treatment. Progressive hyperammonemia can lead to coma, diffuse cerebral edema, and life-threatening neurological injury (1). Here, we report an adult patient with late-onset OTCD whose presenting symptom was acute impaired consciousness. This case highlights the diagnostic difficulty of adult OTCD and demonstrates that multimodal neuromonitoring may serve as an adjunctive tool for continuous assessment of progressive brain injury and for guiding adjustments to clinical management strategies.

## 2 Case report

### 2.1 Patient information

A 34-years-old man was admitted on 3 August 2024 with a 1-day history of vomiting and a 10-h history of altered consciousness. One day before admission, he developed non-projectile vomiting of gastric contents, accompanied by dizziness, blurred vision, and incoherent speech. His symptoms did not improve after treatment at a local hospital. Approximately 10 h before admission, his level of consciousness deteriorated further. He was transferred to our Neurology Department and subsequently to the intensive care unit (ICU) because of generalized convulsions. He had no known underlying medical conditions. However, he reported recent use of butane and methamphetamine and had been hunting outdoors. His father was deceased and had a history suggestive of epilepsy during his lifetime, although it was never formally diagnosed.

### 2.2 Physical examination

On admission to the ICU, a comprehensive physical examination was performed. His temperature was 38.6 °C, heart rate 138 beats/min, respiratory rate 12 breaths/min, and blood pressure 135/84 mmHg. The Glasgow Coma Scale (GCS) score was E1VTM1 (intubated). Both pupils were 4 mm in diameter and reactive to light. Bilateral pathological reflexes were present. There were no signs of bulbar conjunctival edema or meningeal irritation. Limb muscle tone was normal. Cardiovascular, respiratory, and abdominal examinations were otherwise unremarkable. No skin lesions or bite marks were observed.

### 2.3 Laboratory and imaging findings

**FIGURE 1:**
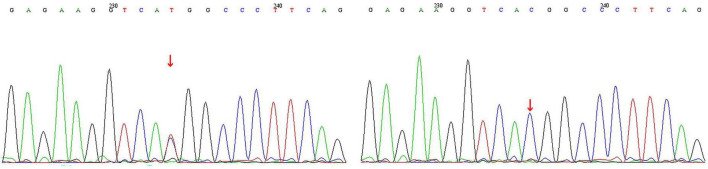
Genetic sequencing of the patient’s mother (left) and sister (right).

Initial laboratory testing revealed marked hyperammonemia (plasma ammonia 478 μmol/L) and an elevated neuron-specific enolase (NSE) level (49 μg/L). Procalcitonin (PCT), C-reactive protein (CRP), cardiac biomarkers, coagulation parameters, and infection-related tests were negative/unremarkable. Cerebrospinal fluid analysis (routine examination and biochemistry) was normal. Toxicology screening was negative, and blood tandem mass spectrometry (MS/MS) did not show a clearly diagnostic amino acid pattern. However, because OTCD was not suspected early in the clinical course, sample collection was delayed and was performed after CRRT had been initiated, which may have reduced the interpretability of this result. The blood acylcarnitine profile showed no obvious abnormalities. Head and neck CT and CT angiography (CTA) showed no obvious abnormalities. Brain diffusion-weighted imaging (DWI) demonstrated symmetric abnormal signals involving the cortex and insula of both cerebral hemispheres. Subsequent Sanger sequencing was performed at an external laboratory. Genetic testing identified a variant in the OTC gene, c.119G > A (p.Arg40His, R40H), in the patient. The testing report classified this variant as “likely pathogenic.” However, available evidence from ClinVar and previous functional studies supports its classification as a pathogenic variant. Further family screening using dried blood spot samples showed that his mother carried the heterozygous variant at the same locus (c.119G > A). In contrast, no variant was detected in his sister (Figure 1).

### 2.4 Treatment

**FIGURE 2:**
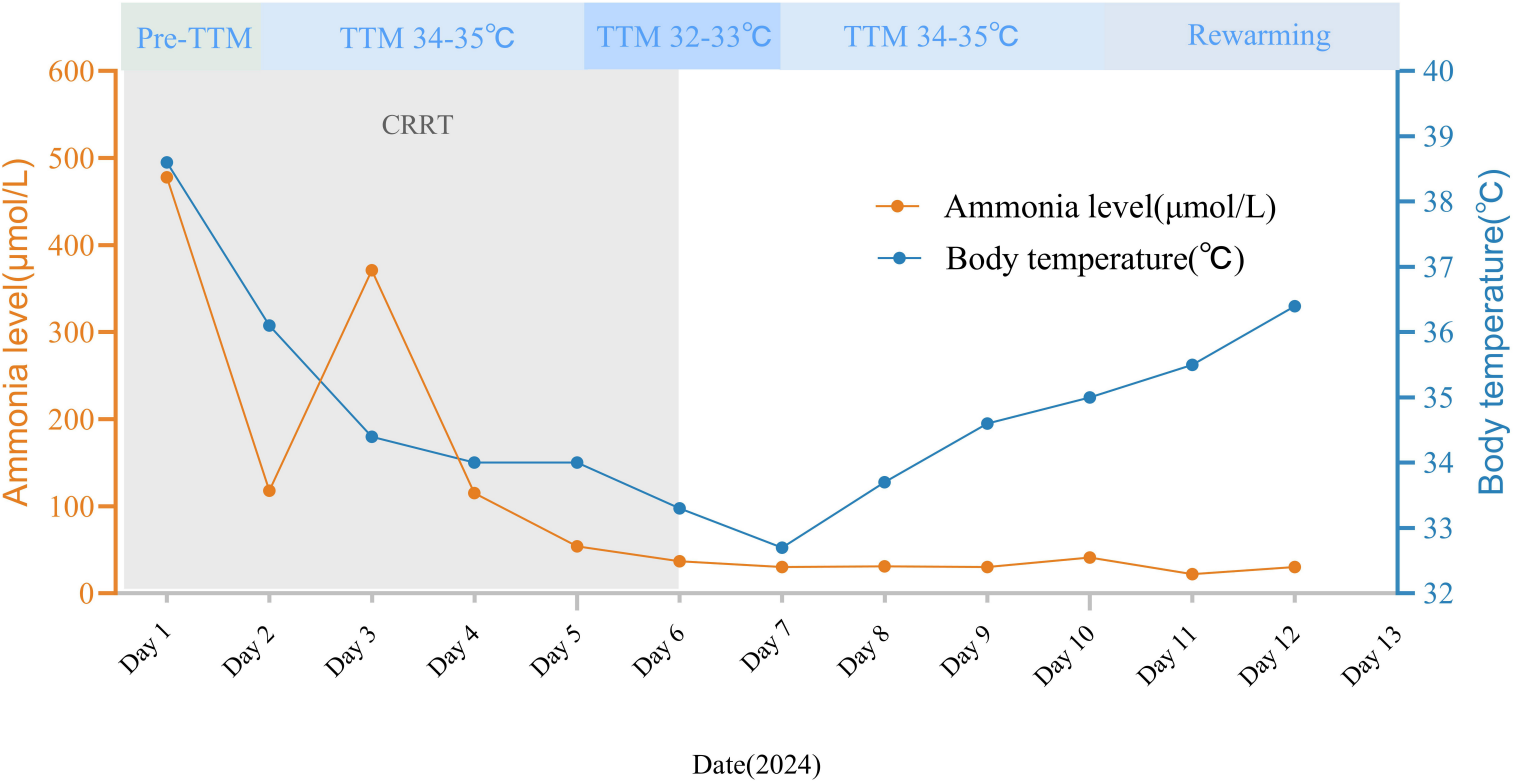
Dynamic changes in blood ammonia level and body temperature during acute management. Serial changes in blood ammonia level and body temperature are shown from Day 1 to Day 12, with Day 1 corresponding to 3 August 2024. The gray shaded area indicates the duration of CRRT from Day 1 to Day 6. The colored strip at the top shows the target temperature-management phases, including pre-TTM, TTM at 34 °C–35 °C, TTM at 32 °C–33 °C, subsequent TTM at 34 °C–35 °C, and rewarming. CRRT, continuous renal replacement therapy; TTM, targeted temperature management.

**FIGURE 3:**
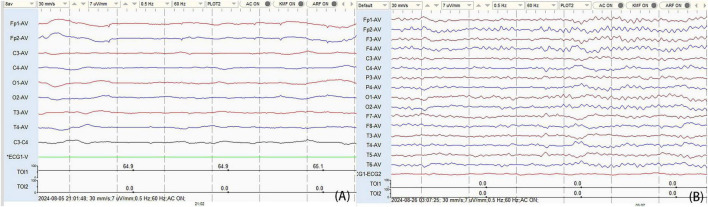
Dynamic EEG. **(A)** Dynamic EEG on day 3 of admission: We provided deep analgesic sedation, and the EEG was markedly suppressed with persistent low amplitude and occasional frontal-dominated δ waves. **(B)** Dynamic EEG on day 24 of admission: after discontinuing the analgesic and sedative drugs, the patient’s consciousness was better than before, and there was a significant increase in fast waves in the EEG background, with a predominantly alpha background.

**FIGURE 4:**
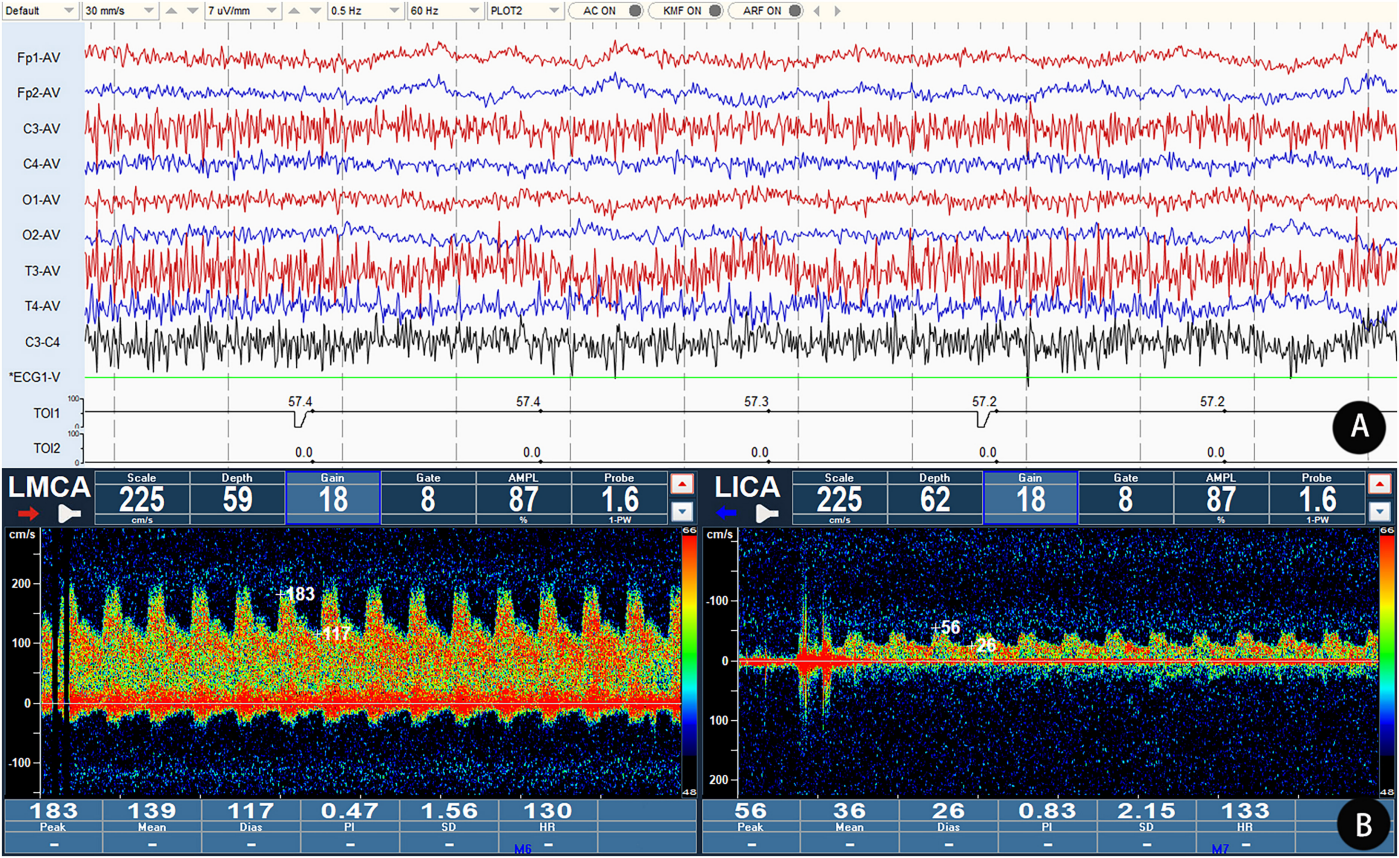
Electroencephalography (EEG) and cerebral blood flow parameters on the fourth day of ICU admission. **(A)** Suspected abnormal discharges on EEG intermittently, accompanied by increased heart rate. Near-infrared spectroscopy (NIRS) showed a value of 57%, slightly decreased from the admission measurement. **(B)** Transcranial Doppler ultrasound revealed markedly increased blood flow velocity in the middle cerebral artery with a low pulsatility index (PI), while the internal carotid artery flow velocity remained within normal limits.

(1) Hyperammonemia management. Given the markedly elevated plasma ammonia level, treatment focused on rapid ammonia removal and restriction of nitrogen intake. Continuous renal replacement therapy (CRRT) was initiated immediately, together with ammonia-lowering pharmacotherapy, including arginine and L-ornithine L-aspartate. Meanwhile, protein and amino acid intake were withheld. Plasma ammonia stabilized by day 4 (Figure 2). (2) Multimodal neuroprotection and neuroprotective management. On ICU admission, head CT suggested diffuse cerebral edema. Osmotic therapy with mannitol and hypertonic sodium chloride solution was administered to reduce cerebral edema. Serial multimodal monitoring was initiated, including transcranial Doppler ultrasonography (TCD), electroencephalography (EEG), near-infrared spectroscopy (NIRS), and bedside ultrasonography. EEG showed marked suppression, and the NIRS value was 65% (Figure 3). On day 2, a repeat head CT indicated further worsening of cerebral edema. TTM was then introduced as an individualized supportive measure, with a core temperature target of 34 °C–35 °C. On day 5, the patient developed tachycardia, with a peak heart rate of 144 beats/min. EEG showed suspected abnormal discharges (Figure 4), TCD revealed markedly increased flow velocity in the middle cerebral artery with a reduced pulsatility index (PI) (Figure 4), and cerebral oxygenation decreased compared with previous measurements. Taken together, these findings raised concern for ongoing cerebral stress and an imbalance between oxygen delivery and consumption. On day 6, the body temperature fluctuated, and the heart rate decreased abruptly. Because multimodal abnormalities persisted, a repeat head CT was performed and showed aggravated cerebral edema. In this context, and as part of broader supportive neurocritical care, the target core temperature was lowered to 32 °C–33 °C to reduce cerebral oxygen consumption and intracranial pressure. By day 10, vital signs gradually stabilized, the EEG showed no abnormal discharges, and TCD suggested improved cerebral hemodynamics. Repeat head CT demonstrated a marked reduction in cerebral edema; cautious rewarming was initiated. (3) Supportive care. Supportive management included mechanical ventilation, anti-infective therapy, antiepileptic therapy, nutritional support, and other ICU supportive measures. Empirical anti-infective therapy was initially administered because severe intracranial infection could not be excluded on admission, but it was discontinued after cerebrospinal fluid examination and infection-related tests showed no evidence of infection.

### 2.5 Prognosis

On day 16, the patient’s GCS score was 6T (E3VTM3), with limited purposeful responsiveness, and we began assisting him to sit for rehabilitation. On day 24, the GCS score improved to 7T (E3VTM4), and the EEG showed a marked increase in fast background activity, predominantly in the alpha range (Figure 3). The patient was then transferred for continued rehabilitation therapy. At telephone follow-up after discharge, the patient was independent in activities of daily living and had relatively preserved cognitive function, but residual emotional disturbance with intermittent irritability persisted. He had reduced protein intake as advised, but repeat blood ammonia testing was not performed because it was unavailable at the local hospital; continued family genetic counseling and metabolic follow-up were recommended.

## 3 Discussion

Ornithine transcarbamylase deficiency is commonly categorized into early-onset (within the first 28 days of life) and late-onset (beyond the neonatal period, ranging from infancy to adulthood) forms. The timing of presentation and disease severity largely depend on residual enzyme activity after the pathogenic variant, as well as environmental and catabolic triggers (3). In the present case, the clinical features were consistent with late-onset OTCD, and genetic testing identified a c.119G > A variant in the OTC gene, supporting the diagnosis.

Late-onset OTCD is frequently misdiagnosed because its initial manifestations are nonspecific. In patients presenting with marked hyperammonemia, OTCD (and other urea cycle disorders) should be considered even in the absence of overt liver failure, particularly when plasma citrulline is low. During acute decompensation, hyperammonemia-related neurotoxicity is the major immediate threat, and plasma ammonia concentrations above 200 μmol/L may lead to coma and respiratory distress (4). Current urea cycle disorder guidelines support early extracorporeal ammonia removal in severe adult hyperammonemia (5). In our patient, prompt CRRT was a central component of initial management.

Targeted temperature management (TTM) is widely used in the ICU because it may reduce cerebral metabolic demand, attenuate the post-injury inflammatory response, decrease cerebral edema, lower intracranial pressure, and provide neuroprotection (6). However, these potential neuroprotective effects have primarily been observed in other neurocritical care settings, and current guidelines for urea cycle disorders do not recommend TTM as a standard treatment for hyperammonemic encephalopathy (5). Previous studies have suggested that when conventional measures provide inadequate control of cerebral edema and intracranial pressure (ICP), induced hypothermia at 32 °C–35 °C may be considered to help control cerebral edema and ICP, with maintenance for several days followed by slow rewarming (7). In this patient, intracranial conditions remained difficult to control despite active pharmacological treatment. Therefore, targeted temperature management was implemented, and the target core temperature was dynamically adjusted with the aim of limiting cerebral edema. A previous case report also described a similar application of TTM in late-onset OTCD with favorable neurological recovery (8). However, this evidence remains limited to the case-report level. The patient’s clinical improvement was likely the result of multiple concurrent therapeutic interventions, and the use of targeted temperature management in this condition requires further validation in future clinical studies. In addition, the potential risks of TTM, including arrhythmia, infection, coagulopathy, electrolyte disturbance, shivering, immunosuppression, and altered drug metabolism, should be carefully weighed against its uncertain benefits in OTCD-related hyperammonemic encephalopathy.

This case was clinically complex. Although plasma ammonia normalized by hospital day 4, the patient subsequently developed marked fluctuations in vital signs accompanied by episodic muscle activity, raising concern for ongoing secondary brain injury despite biochemical improvement. This temporal dissociation between normalization of peripheral blood ammonia levels and worsening cerebral edema or neurological function is consistent with previous observations. Relevant studies suggest that this phenomenon may be related to delayed clearance of glutamine within the brain and a possible “osmotic trap,” whereby local cytotoxic cerebral edema and brain stress may persist even after peripheral ammonia has been cleared (9). In this setting, multimodal neuromonitoring provided serial bedside information that was considered alongside clinical status and neuroimaging. Worsening CT-defined cerebral edema contributed to the decision to initiate TTM. On day 5, tachycardia, reduced NIRS values, and increased TCD flow velocities suggested persistent cerebral stress and possible oxygen supply-demand mismatch. On day 6, persistent multimodal abnormalities prompted repeat neuroimaging, which confirmed worsening cerebral edema; these findings were considered together when deciding to further lower the target temperature. Subsequent improvements in the EEG background, TCD parameters, and CT findings were used to support cautious rewarming. Based on these observations, we speculate that, in addition to supporting individualized management during the acute phase, multimodal neuromonitoring may continuously capture dynamic changes in cerebral blood flow, cerebral oxygen metabolism, and electrophysiological activity. In this way, it may provide a non-invasive and practical means of monitoring the evolution of central nervous system injury after biochemical parameters have normalized, thereby helping guide subsequent adjustments in clinical decision-making. Differential considerations for the delayed neurological instability included seizures, paroxysmal sympathetic hyperactivity (PSH), and ongoing delayed brain injury after the hyperammonemic phase. Seizures at presentation could not be completely excluded, given the generalized convulsions at ICU admission and the known association between severe hyperammonemia and seizure risk (10, 11). However, continuous EEG did not demonstrate definitive epileptiform discharges, and interpretation was limited by artifacts and concurrent antiseizure and sedative therapy. PSH was also considered because episodic hyperthermia, tachycardia, and motor activity were observed (12), but the diagnosis remained presumptive. More broadly, delayed progression of cerebral edema after ammonia normalization may provide a unifying explanation for this clinical course, as fatal cases with persistent coma despite biochemical correction have been reported (13). In the setting of overlapping clinical manifestations and limited neurological examination due to sedation, multimodal neuromonitoring showed potential value for differential diagnosis. Cross-comparison of TCD, NIRS, and EEG findings may provide additional information for distinguishing occult non-convulsive epileptiform activity, autonomic instability, and delayed impairment of cerebral perfusion.

## 4 Conclusion

Late-onset OTCD is difficult to diagnose because of its rarity and nonspecific presentation. The possibility of OTCD should be considered in adults with acute encephalopathy and marked hyperammonemia, even when overt liver failure is absent. Early recognition, rapid ammonia removal, and comprehensive ICU support remain the cornerstone of management. In addition, normalization of blood ammonia levels in these patients does not necessarily indicate resolution of the central nervous system crisis, and neurological deterioration may still continue. In this context, serial multimodal neuromonitoring may provide additional bedside information beyond routine clinical examination, particularly when neurological assessment is limited by sedation. This may support timely recognition of patients at risk of ongoing secondary brain injury and assist in reassessment of individualized neuroprotective strategies. However, the general clinical value of this monitoring approach, as well as the safety and ultimate efficacy of TTM in this setting, remains uncertain and requires further investigation in future studies.

## Data Availability

The original contributions presented in this study are included in this article/supplementary material, further inquiries can be directed to the corresponding author.
